# One-Pot Preparation of Hydrophilic Polylactide Porous Scaffolds by Using Safe Solvent and Choline Taurinate Ionic Liquid

**DOI:** 10.3390/pharmaceutics14010158

**Published:** 2022-01-10

**Authors:** Anna Clara De Felice, Valerio Di Lisio, Iolanda Francolini, Alessia Mariano, Antonella Piozzi, Anna Scotto d’Abusco, Elisa Sturabotti, Andrea Martinelli

**Affiliations:** 1 Department of Chemistry, Sapienza University of Rome, P.le Aldo Moro 5, 00185 Rome, Italy; annaclara.defelice@gmail.com (A.C.D.F.); valerio.dilisio@dipc.org (V.D.L.); iolanda.francolini@uniroma1.it (I.F.); antonella.piozzi@uniroma1.it (A.P.); 2 Department of Biochemical Sciences, Sapienza University of Rome, P.le Aldo Moro 5, 00185 Rome, Italy; alessia.mariano@uniroma1.it (A.M.); anna.scottodabusco@uniroma1.it (A.S.d.)

**Keywords:** polylactide, porous scaffold, tissue engineering, ethyl acetate, choline taurinate, ionic liquid

## Abstract

Polylactides (PLAs) are a class of polymers that are very appealing in biomedical applications due to their degradability in nontoxic products, tunable structural, and mechanical properties. However, they have some drawbacks related to their high hydrophobicity, lack of functional groups able to graft bioactive molecules, and solubility in unsafe solvents. To circumvent these shortcomings, porous scaffolds for tissue engineering were prepared by vigorously mixing a solution of isotactic and atactic PLA in nontoxic ethyl acetate at 70 °C with a water solution of choline taurinate. The partial aminolysis of the polymer ester bonds by taurine –NH_2_ brought about the formation of PLA oligomers with surfactant activity that stabilized the water-in-oil emulsion. Upon drying, a negligible shrinking occurred, and mechanically stable porous scaffolds were obtained. By varying the polymer composition and choline taurinate concentration, it was possible to modulate the pore dimensions (30–50 µm) and mechanical properties (Young’s moduli: 1–6 MPa) of the samples. Furthermore, the grafted choline taurinate made the surface of the PLA films hydrophilic, as observed by contact angle measurements (advancing contact angle: 76°; receding contact angle: 40°–13°). The preparation method was very simple because it was based on a one-pot mild reaction that did not require an additional purification step, as all the employed chemicals were nontoxic.

## 1. Introduction

Polylactides or polylactic acids (PLAs) are one of the most studied and employed synthetic polymer classes in many medical specialties, including orthopedic, general and plastic surgery, dentistry, and pharmacology. The market offers a wide variety of PLA-based commercial devices [1,2], and a very wide range of scientific literature suggests PLAs for the preparation of temporary prostheses and devices, drug-delivery systems, and scaffolds for tissue engineering as well. This results from the combination of the typical advantages of synthetic polymers, such as tunable chemical and physical properties, and PLAs’ specific features. In fact, by selecting the proper ratio between the two enantiomeric monomers in the polymerization feed, isotactic poly(L-lactide) (PLLA) and poly(*D*-lactide) (PDLA) or completely atactic poly(*DL*-lactide) (PDLLA) polymer, as well as polymers with different *D-L* contents, can be obtained. Therefore, according to their composition, PLAs’ crystallinity and chemical, physical, thermal, and mechanical properties are easily modulated. Moreover, PLAs are biocompatible and biodegradable, and the degradation rate can be tuned by crystallinity. In body tissues, PLAs are resorbed with small or negligible adverse response, and are approved by national and international agencies (FDA, EMA) for medical uses [3]. However, polylactides have some drawbacks that, in some specific applications, must be circumvented. As far as the processing is concerned, stereo-regular polymers are soluble in chlorinated or other toxic solvents, such as aromatic liquids, THF, or dioxane. Then, in the transformation process involving polymer solutions, besides environmental and worker safety concerns, the elimination of solvent traces is mandatory [4]. Moreover, in application where a high specific surface area is needed, such as in the production of micro- or nanoparticles for drug-delivery systems and porous scaffolds for tissue engineering, the use of surfactants is necessary to stabilize suspensions, emulsions, or foams. These chemicals could be toxic or could interfere negatively with the drug-delivery kinetics and biodistribution, nanoparticle intracellular uptake, or cell adhesion. In addition, the highly hydrophobic character of the polymer brings about some negative consequences, such as particle aggregation in body fluids and obstacle adhesion, growth, and migration of cells into the scaffolds [5,6]. Finally, PLAs do not have functional groups able to link bioactive molecules necessary to improve cell–material interactions. Despite this, the general PLA features are so suitable in many applications that, rather than use other polymers, numerous different measures have been taken to overcome the above-mentioned limits. These can be divided into two general strategies, adopted in the polymer’s synthesis or in the polymer’s postmodification stage. Regarding the first point, lactide ring-opening polymerization (ROP) [7] has been used to synthetize block [8,9,10], graft [11,12], or brush [13] copolymers initiated from hydroxyl or amine groups of hydrophilic comonomers. Among ROP initiators, PEG is the most employed. PEG-b-PLA copolymers, in fact, can self-assemble in micelles or gels with tunable properties, according to the molecular weights and polymer structures of the PEG and PLA. Alternatively, PLAs with a self-emulsifying character, composed of the hydrophobic polymer tail and polar head, have been obtained for micro- and nanoparticles, or scaffold preparation without external surfactant. Carboxylate [14], carbohydrates [15], or l-α-glycerophosphorylcholine [16] are some examples of moieties that have been used as hydrophilic terminal groups. Polymer surface modification is the second strategy used to improve the hydrophilicity of PLA by introducing functional groups able to interact specifically with bioactive molecules that favor the polymer–cell interaction. It is based on physical (plasma treatment [17,18,19], ion implantation [20], or UV radiation [21]) or wet chemical methods [22,23], or a combination of them [24,25,26]. Controlled hydrolysis by acid or basic treatments, or aminolysis with di- or multifunctional amines, provide the PLA surface with hydroxyl [27], carboxylic [28], or primary or secondary amine groups [29,30,31] able to increase hydrophilicity, while favoring the absorption of proteins or charged polysaccharides through electrostatic interaction, as well as preparing the polymer surface for further grafting reactions. In a previous paper, we introduced sulfonic groups directly on a PLLA film surface through an aminolysis reaction carried out by taurine (Tau) [32]. Differently from widely used di- or multifunctional amines, such as ethylene diamine or hexamethylene diamine, taurine is a nontoxic and safe physiological amino acid produced by mammalian cells. Moreover, with respect to carboxylic groups, the sulfonic group on the polymer surface exists in a dissociated anionic form also at low pH, allowing the electrostatic interaction with positively charged proteins or polysaccharides in a wide pH range condition. It has been observed that Tau grafting and collagen adsorption on PLLA films improved human primary chondrocyte adhesion and growth. In order to obtain a complete solubilization in the reaction medium (methanol) and to avoid the zwitterion formation, it has been necessary to transform Tau in its salt with tetrabutylammonium (TBA). In addition, traces of TBA remains adsorbed on films surface after the exchange with Na^+^ ions, leading to possible negative long-term effects on cell viability. 

In this paper, we report a method for the preparation of PLA-based porous scaffolds designed to take into account the possible solutions to the aforementioned drawbacks. To this end, a water-in-oil emulsion drying method was used. In particular, a water solution of the nontoxic ionic liquid choline taurinate was added as a porogen dispersed phase to a mixture of PLLA and PDLLA solubilized in ethyl acetate at high temperature. The low-molecular-weight PLA chains that resulted from the partial in situ aminolysis of the polyester, acted as surfactants and stabilized the emulsion. The advantages of the proposed method were that all the chemicals were moderately or nontoxic, the one-pot procedure was simple, the purification was not mandatory, and the porosity and pore dimensions of the porous scaffolds could be easily tuned by changing the emulsion composition. 

## 2. Materials and Methods

Poly(*L*-lactide) (PLLA) Ingeo Biopolymer 3251D from NatureWorks (Mw = 90 kg × mol^−1^, Mn/Mw = 1.6) was purified by crystallization at room temperature starting from an ethyl acetate solution (5 wt %) at 70 °C.

Medical grade poly(*D,L*-lactide) (PDLLA) PURASORB^®^ PDL 02A (η_inh_ = 0.16–0.24 dL × g^−1^ in chloroform at 25 °C, c = 1 g × dL^−1^) was kindly supplied by Corbion and used as received.

Collagen (Atelocollagen sponge) was purchased from Cosmo Bio Co. (Tokyo, Japan).

All other chemicals were purchased from Sigma-Aldrich and used as received.

### 2.1. Choline Taurinate Preparation

The ionic liquid choline taurinate ([Ch][Tau]) was prepared from a modification of the recipe described by Azizi et al. and Deng et al. [33,34]. First, choline hydroxide (ChOH) was prepared mixing an equimolar amount of choline chloride and KOH in methanol (1 mmol per 1.5 mL) at 60 °C for 3 h. Then, solid KCl was filtered, and taurine in water (20 mol% excess) was immediately added to the methanolic solution of ChOH, due to the susceptible nature of choline hydroxide. The solution was stirred for 3 h at room temperature and then dried under vacuum at 40 °C. The [Ch][Tau] ionic liquid was dissolved in methanol, and the excess of taurine was removed by filtration. Lastly, the alcoholic solution was dried under vacuum, and [Ch][Tau] was obtained as a transparent viscous ionic liquid (yield 85%).

### 2.2. Aminolysis Reaction in MeOH

Preliminary experiments were carried to assess the surfactant property of the products of the PDLLA aminolysis by [Ch][Tau]. The reaction was carried out by adding a weighted amount of PDLLA to a solution of ionic liquid in MeOH (5 mg/mL) in such quantity to have 1 mole of [Ch][Tau] per 10 moles of polymer-repeating units. After 2 h at 60 °C under stirring, the reaction products dissolved completely. The solution was dried under vacuum, and then ethyl acetate (EtOAc) was added. The EtOAc insoluble fraction, separated by centrifugation, was soluble in water. Both the EtOAc and water-soluble products were dried and characterized by FTIR. The surfactant activity of the two fractions, as well as of the [Ch][Tau], was evaluated by vigorously shaking a mixture of 1 mL of water and 1 mL of EtOAc with 50 mg of each product. After 5 min, the possible separation of the liquid phases was controlled visually.

### 2.3. Aminolysis Reaction of PLA Film in Water

The aminolysis reaction was also carried out in water on PLA films. They were prepared by slow evaporation of a chloroform solution of PLLA+PDLLA mixture in Petri dishes in the different compositions reported in Table 1. The dried films, of about 100 μm thickness, were soaked in a 5 wt/v % solution of [Ch][Tau] for 2 h at 60 °C. Then, the sample were rinsed with distilled water and dried. The samples were characterized by ATR-FTIR and dynamic contact angle analyses.

### 2.4. Scaffold Preparation and Collagen Absorption

The scaffolds were prepared by an emulsion freeze-drying method. The PDLLA was completely soluble in EtOAc at room temperature, while the PLLA dissolved at 70 °C. Therefore, an 8 wt/v % solution of PLLA or of a mixture of PDLLA and PLLA was obtained at 70 °C in a glass test tube. Then, the organic phase was mixed by a homogenizer at 1500 rpm, and an equal volume of [Ch][Tau] water solution was added dropwise in order to avoid rapid cooling and polymer precipitation. After 10 min, the stable emulsion was cooled by immersing the test tube in water at 15 °C to favor the solution gelation. The scaffold was obtained by removing the solvents by vacuum drying without any purification step. The employed PLLA weight fractions in the polymer blend (PLLA+PDLLA) and [Ch][Tau] solution concentrations, as well as the sample codes, are reported in Table 1.

The presence of the anionic −SO_3_^−^ groups of the taurine grafted on the scaffold surface was exploited to absorb collagen through electrostatic interaction. The scaffolds were dwelled in an acidic water solution (pH = 3) of the protein (0.1 wt/v %) for 2 h at room temperature and then rinsed in distilled water for 6 h, with changing of the water every hour.

All the prepared scaffolds were characterized by ATR-FTIR and morphological analyses, as well as by mechanical tests.

### 2.5. Characterization

#### 2.5.1. FTIR Analysis

The [Ch][Tau] ionic liquid, the EtOAc and water soluble fractions of PDLLA aminolyzed in methanol, as well the scaffolds were characterized by FTIR in attenuated total reflection mode (ATR) by using a Thermo Nicolet 6700 instrument (Thermo Scientific, Waltham, MA, USA), equipped with a Golden Gate diamond single-reflection device (Specac LTD, Orpington, UK). The ATR-FTIR spectra were collected using 200 scans in the range of 4000–650 cm^−1^ at a resolution of 4 cm^−1^.

#### 2.5.2. Dynamic Contact Angle Analysis

The dynamic contact angle of the PLA films, before and after the reaction with [Ch][Tau] water solution, was measured in water at room temperature by a dynamic contact angle analyzer (DCA-312, Chan, Cerritos, CA, USA) at a stage speed of 40 μm × s^−1^. The results are reported as mean value ± standard deviation evaluated on three repetitions.

#### 2.5.3. Porosity Determination

The total porosity *p_t_*; i.e., the pore/material volume ratio, was experimentally measured from the apparent density of the scaffolds *ρ_sc_* = *m*/*V_sc_*, where *m* is the weight and *V_sc_* is the geometric volume of the porous scaffolds, according to the equation:

(1) $$p_{t}=\left(1-\frac{\rho_{sc}}{\rho_{PLA}}\right)\times 100$$

where *ρ_PLA_* = 1.29 g cm^−3^ is the density of the PLA.

The open porosity (*p_o_*), which accounted only for the scaffold void volume accessible to water (i.e., the interconnected pores), was determined by soaking the sample in water; *p_o_* was calculated using the equation:

(2) $$p_{o}=\frac{V_{l}}{V_{sc}}\times 100$$

where *V_l_* is the accessible (open) pore volume, evaluated from the increase in the scaffold weight after swelling, divided by the soaking liquid density.

#### 2.5.4. Morphological Characterization

The morphology of the scaffolds was investigated by scanning microscopy using a field emission scanning electron microscope (AURIGA, Zeiss, Jena, Germany). The pore diameters and their distribution were evaluated by measuring 250 pores per image using Gwyddion 2.53 software. Mean pore dimension and standard deviation were derived by assuming a log-normal distribution.

#### 2.5.5. Characterization of Mechanical Properties 

Uniaxial compression tests on the scaffolds were carried out at RT using an INSTRON 4502 instrument (Instron Inc., Norwoon, MA, USA). The samples, cut in a parallelepiped shape (base dimensions of about 5 × 5 mm^2^, height of about 8 mm), were placed between the two flat plates and compressed at 5 mm min^−1^ using a 10 N load cell. The Young’s moduli and 2% offset yield strength were determined from stress–strain curves obtained by reporting the apparent stress *σ* = F/A (MPa), where F is the compression force and A is the initial cross-sectional area of each test specimen, versus the strain *ε* = (*L*_0_ − *L*)/*L*_0_, where *L*_0_ and *L* are the initial and the deformed sample heights, respectively. In particular, the Young’s modulus was calculated from the slope of the initial linear zone in the stress–strain curve. The results are reported as average values ± standard deviations on at least three experiments conducted on different specimens.

#### 2.5.6. Assessment of Choline Taurinate Cytotoxicity

Choline taurinate cytotoxicity was analyzed by culturing human primary chondrocytes (HPCs), isolated from femoral and tibial condyles and from femoral heads, that were obtained from patients who underwent a total knee and hip arthroplasty. Full ethical consent was obtained from all donors, and the study was approved by the Research Ethics Committee, Sapienza University of Rome. First, 2 μL, 5 μL, or 10 μL of [Ch][Tau] was added in the well of a tissue culture plate, each containing 8 × 10^3^ cells, conditioned in Dulbecco’s Modified Eagle’s Medium DMEM without red phenol supplemented with L-glutamine, penicillin/streptomycin, gentamycin, and 10% fetal bovine serum (FBS) for 48 h at 37 °C, in 95% humidity and a 5% CO_2_ atmosphere. Cells cultured in the absence of [Ch][Tau] were used as the control (CTL). Cellular viability was quantified by measuring the mitochondrial dehydrogenase activity using a colorimetric assay based on tetrazolium dye 3-(4,5-dimethylthiazol-2-yl)-5-(3-carboxymethoxyphenyl)-2-(4-sulfophenyl)-2H-tetrazolium (MTS, Promega Corporation, Madison, WI, USA), according to the manufacturer’s instructions. Briefly, after 48 h, 10% (*v*/*v*) of MTS dye was added to the culture media, and cells were cultured for 3 h to allow the formation of soluble formazan crystals by viable cells. Spectrophotometric absorbance was measured at 490 nm using an Appliskan multiplate reader (Thermo Fisher, Waltham, MA, USA). The results are reported as average values ± standard error calculated for at least three experiments.

## 3. Results

A solution of PLLA, PDLLA, or a mixture of them in EtOAc at 70 °C did not form a stable suspension when vigorously mixed with water, and the phase separation occurred in a few seconds when the stirring was stopped. In the absence of polymers, neither [Ch][Tau] in water brought about the formation of a suspension, meaning that the ionic liquid did not have surfactant activity. Conversely, a stable suspension could be obtained by thoroughly shaking the solutions of polymer in EtOAc and [Ch][Tau] in water for almost 10 min at high temperature. This behavior attributed to possible surfactant activity of the polymer and the ionic liquid reaction product, and thus, preliminary experiments were carried out to investigate the observed phenomena. 

**Figure 1:**
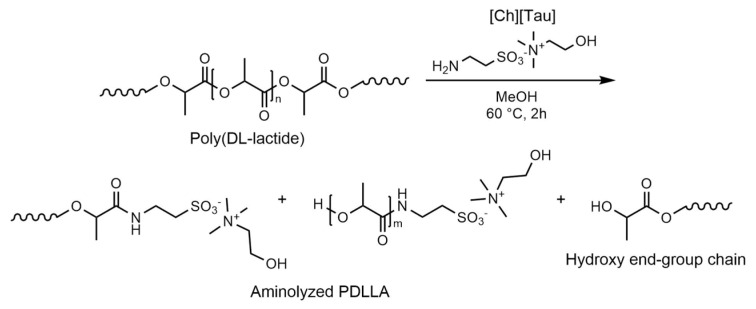
Scheme of the reaction pathway for the aminolysis of PDLLA in methanol in the presence of choline taurinate. The amidated products characterized by higher molecular weight (high *m*) were soluble in EtOAc, while shorter oligomers (low *m*) solubilized in water. Chains with hydroxy end-group were obtained as the result of aminolysis at the carbonyl group.

In a previous paper, the heterogeneous aminolysis of a solid PLLA film by the tetrabutylammonium salt of taurine in methanol was carried out in a controlled mild condition, 40 °C for 60 min, to avoid extensive film erosion [32]. The grafting of taurine on the polymer surface was ascertained by XPS, ATR-FTIR, and morphological SEM analyses. In the present research, harsher conditions were used to achieve extensive aminolysis of PDLLA in methanol (reaction scheme in Figure 1), to the point that the aminolyzed products dissolved in the reaction medium.

**Figure 2:**
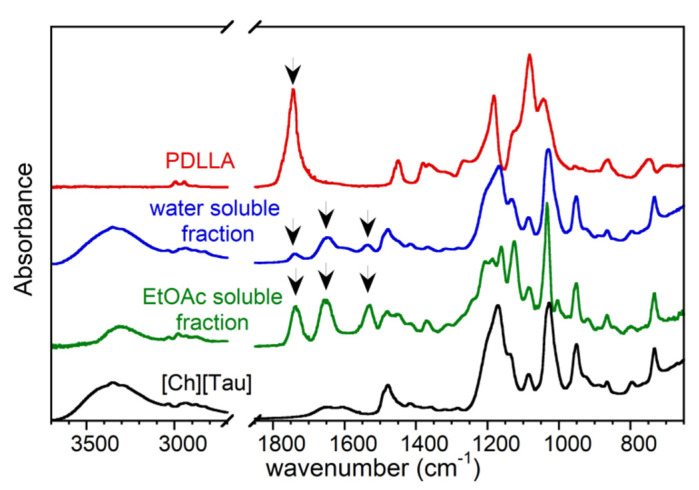
FTIR spectra of poly(*DL*lactide) (PDLLA), choline taurinate ([Ch][Tau]), and water-soluble and ethyl-acetate-soluble reaction products. The arrow at 1740 cm^−1^ indicates the C=O stretching absorption of ester bond; the arrows at 1650 cm^−1^ and 1530 cm^−1^ indicate the absorptions of I and II amide bands, respectively.

At the end of the reaction, the recovered products were soluble in EtOAc or in water. The ATR-FTIR spectra of the two fractions are displayed in Figure 2. For the sake of comparison, the spectra of the virgin amorphous PDLLA and [Ch][Tau] are also reported.

The [Ch][Tau] spectrum was characterized by the large absorption between 3360 cm^−1^ and 3060 cm^−1^ due to the O–H and N–H stretching; the bands at 1026 cm^−1^ and 1168 cm^−1^ were attributed to the −SO_3_^−^ symmetric and asymmetric stretching, respectively; and the band at 953 cm^−1^ was assigned to ammonium C–N^+^(CH_3_)_3_ asymmetric stretching of choline [35,36].

As a consequence of the reaction, the spectra of the aminolyzed product showed the weakened C=O stretching band of the PDLLA ester bond at about 1740 cm^−1^ and the appearance of the amide I (C=O stretching) and II (N–H bending + C–N stretching) absorptions at 1650 cm^−1^ and 1530 cm^−1^, respectively, as well as the characteristic bands of [Ch][Tau]. The degree of amidation of PDLLA could be inferred from the comparison between the ester and amide absorptions. In the spectra of the fraction soluble in water, the intensity of the ester C=O stretching band was weaker than those of amides, a sign of low-molecular-weight aminolysis products (low *m* in Figure 1). On the other hand, PDLLA oligomers with a higher molecular weight (high *m* in Figure 1), characterized by a comparable intensity of ester and amide group signals, resulted were in EtOAc (EtOAc soluble fraction in Figure 2).

Each of the two aminolysis products were vigorously shaken in a 1:1 *v*/*v* EtOAc:water mixture. After the stirring was stopped, the mixture with a water-soluble fraction formed an unstable suspension of organic liquid in water (o/w) that tended to demix after 3 min. Conversely, the mixture with an EtOAc-soluble fraction brought about a stable suspension of water in the organic phase (w/o). Therefore, it was possible to infer that the aminolysis reaction between the polymer dissolved in EtOAc and [Ch][Tau] in water at 60 °C led to the formation of polylactide oligomers provided with a hydrophilic head with surfactant activity that favored the w/o suspension. 

**Figure 3:**
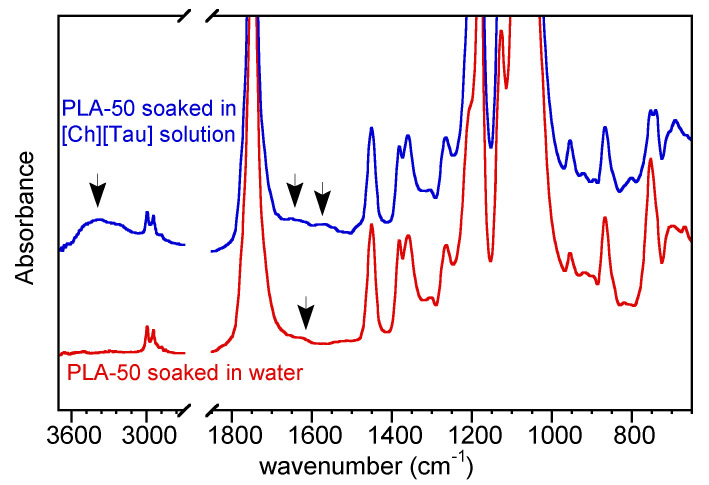
FTIR spectra of PLA-50 soaked in water and in [Ch][Tau] water solution for 10 min at 70 °C. The arrow at 1620 cm^−1^ indicates the carboxylic C=O stretching absorption of the sample soaked in water due to a light hydrolysis reaction. The arrows at 1650 and 1550 cm^−1^ indicate the two bands of amide I and II and, at 3400 cm^−1^, the absorption of N-H and O-H stretching of the sample soaked in [Ch][Tau] solution.

The aminolysis of polyester is a widely described reaction mostly carried out in an alcoholic medium, although some papers reported the use of an amine water solution [31,37]. Then, the reaction of [Ch][Tau] with polylactide in water was preliminarily investigated in a heterogeneous liquid/solid condition. Films obtained by casting a solution of PLLA or a PLLA/PDLLA mixture in chloroform were soaked in water as a control, and in the ionic liquid solution in water (1% wt/v) at 70 °C for 10 min. In Figure 3, the ATR-FTIR spectra of the control and aminolyzed PLA-50 film are compared. 

The sample soaked in water showed a small peak at 1620 cm^−1^ due to carboxylic C=O stretching, indicative of a light hydrolysis reaction, while the film treated with [Ch][Tau] solution was characterized by the two bands of amide I and II at about 1650 cm^−1^ and 1550 cm^−1^, respectively, as well as the broad absorption of N–H and O–H stretching at about 3400 cm^−1^.

The aminolysis reaction involved changes in surface hydrophilicity that were investigated by contact angle measurements in water. 

**Figure 4:**
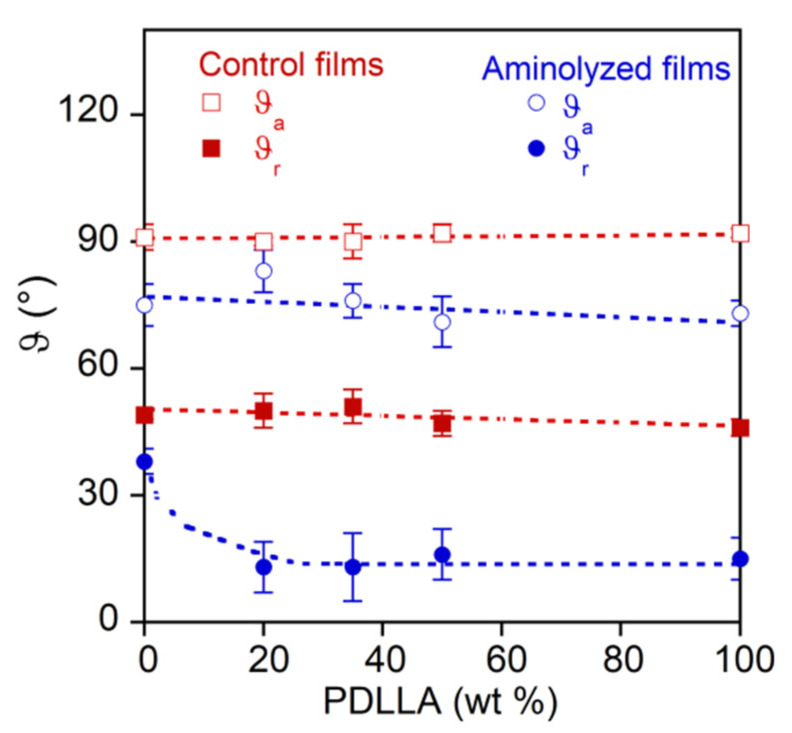
Advancing and receding contact angles of pristine and reacted PLA films as a function of PDLLA weight fraction.

In Figure 4, the receding (*ϑ_r_*) and advancing (*ϑ_a_*) contact angle of the polymer films before and after the aminolysis reaction are reported as a function of the PDLLA weight fraction.

Figure 4 shows that the advancing and receding contact angle did not vary with the composition of pristine samples, and evidenced the hydrophobic nature of the PLAs, characterized by *ϑ_a_* of about 91°. As a result of the aminolysis reaction, the advancing contact angle was lightly affected by the film composition, and it decreased at a mean value of about 76°. Similar results have been reported for the reaction of PLLA with tetrabutylammonium salt of taurine carried out in methanol [32]. The receding contact angle was more influenced by aminolysis and film composition, decreasing from about 40° of PLLA to 13° by adding 20 wt % of PDLLA, and then nearly levelling up at the higher PDLLA content. This behavior can be explained by the different reactivities of crystalline PLLA, which was more stable, and amorphous PDLLA, which was more susceptible to aminolysis. The advancing contact angle, related to the less wettable surface fraction, was associated with the lightly reacted PLLA fraction, while the receding contact angle was associated with the hydrophilic surface portion of the aminolyzed PDLLA. 

As reported in the Materials and Methods section, porous scaffolds were prepared by solubilizing PLLA or a PLLA/PDLLA mixture at different weight ratios in EtOAc at 70 °C, and by adding the [Ch][Tau] water solution under vigorous stirring. Then, after obtaining the suspension, the cooling of the test tube to about 15 °C led to the formation of a physical gel, due to the precipitation of PLLA at low temperature.

However, the gel obtained using only PLLA was very fragile, and broke apart when extracted from the test tube in wet or dry conditions. This was due to the crystallization of the isotactic polymer in the form of poorly cohesive lamellar crystallites.

For this reason, atactic PDLLA was added to the PLLA solution to prevent the crystallization and enable a continuous structure formation. In this way, the obtained physical gels were more stable, and underwent negligible shrinking upon drying. 

**Figure 5:**
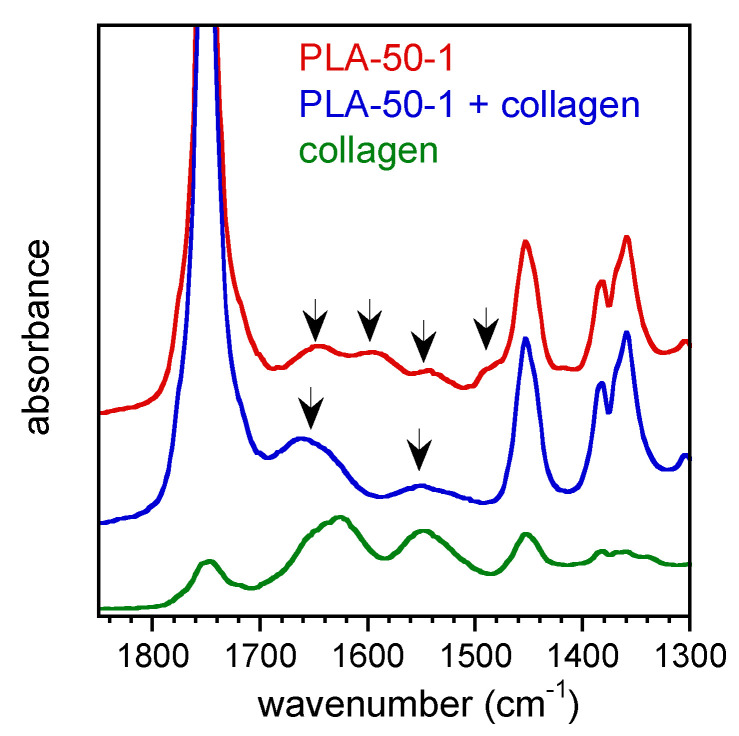
FTIR spectra of PLA-50-1 scaffold, before and after the absorption of collagen, and of collagen. The arrows at 1550 cm^−1^ and 1650 cm^−1^ indicate the amide I and II bands, respectively; and at 1600 cm^−1^ and 1480 cm^−1^, the absorption of [Ch][Tau] of the PLA-50-1 sample. The arrows at 1650 cm^−1^ and 1550 cm^−1^ highlight the bands of collagen adsorbed on PLA-50-1 sample after the dwelling in the protein solution.

The scaffolds were characterized by ATR-FTIR analysis before and after the absorption of collagen. As an example, the FTIR spectra of the PLA-50-1 scaffold and of the protein are reported in Figure 5. 

The spectrum of the PLA-50-1 scaffold showed, besides the absorption of PLA between 1700 cm^−1^ and 1450 cm^−1^, four additional bands: two at about 1550 cm^−1^ and 1650 cm^−1^, due to the amide I and amide II, respectively, which were already observed in the spectra of the aminolyzed film (Figure 3); and two others at 1600 cm^−1^ and 1480 cm^−1^ due to [Ch][Tau], the spectrum of which is reported in Figure 3. Differently from the reacted films, the presence of the ionic liquid was well visible in all the scaffolds because they were not rinsed after the preparation, and due to their large surface area. As a result of collagen absorption, the scaffold spectrum showed the two characteristic large bands of peptide bonds at 1650 cm^−1^ and 1550 cm^−1^, confirming the stable interaction of the protein with the anionic polymer surface, even after repeated rinsing with distilled water.

The total (*p_t_*) and open porosity (*p_o_*) were evaluated using Equations (1) and (2), and the results are reported in Table 1. All the samples, independently from the preparation conditions, showed a similar porosity, and the small difference between *p_t_* and *p_o_* values indicated that the pores were highly interconnected; that is, they were all accessible to water infiltration. Moreover, the similarity of the measured and expected porosities, evaluated while taking into account the ratio between the solid and liquid fractions used in scaffold preparation (expected porosity 97%), evidenced that all the samples were subject to a negligible shrinking upon drying. This observation allowed us to presume that the porosity could be modulated by varying, for instance, the added water solution volume, a procedure not yet investigated. 

**Figure 6:**
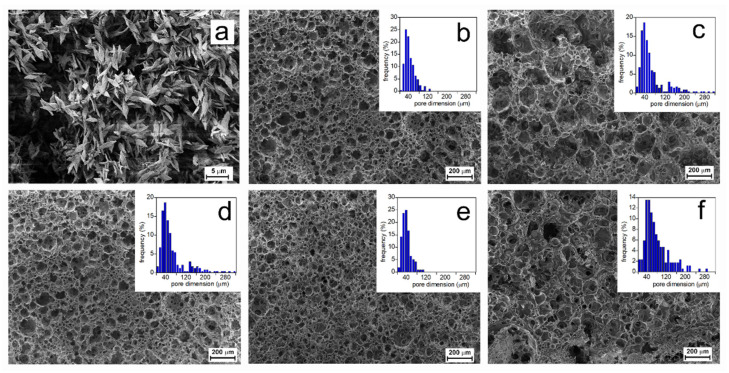
SEM micrographs of the scaffolds prepared by varying polymer mixture composition and [Ch][Tau] concentration in water: (**a**) sample composed of PLLA only; (**b**) PLA-50-1; (**c**) PLA-65-0.32; (**d**) PLA-65-1; (**e**) PLA-65-5; (**f**) PLA-80-1. In the insets, the histograms of pore diameter distributions are reported.

The dried porous scaffolds were cut with a scalpel blade, and the morphology was analyzed by SEM (Figure 6). In Figure 6, the insets report the histograms of the pore diameter distributions from which the mean pore diameters were calculated (Table 1).

The images show that the scaffold pores were mostly interconnected, as expected from porosity measurements. In addition, it was observed that the morphology was affected by the composition of the suspensions. The PLA-65-0.32 and PLA-50-1 samples showed a double pore distribution, while the PLA-80-1 and PLA-65-1 scaffolds had a wide distribution, as displayed in the Figure 6 insets. The pore dimensions approximately followed a log-normal distribution, from which the mean diameters and standard deviations were evaluated (reported in Table 1). In general, the mean pore dimension increased by increasing the PDLLA content and by decreasing the [Ch][Tau] concentration. Since PDLLA is characterized by a high molecular weight, it was presumed that the polymer solution viscosity increased by increasing the atactic polymer concentration, disfavoring the coalescence of the suspended larger water drops. As far as the effect of [Ch][Tau] concentration, its increase brought about an increase in aminolyzed surfactant-like oligomers, and then the stabilization of a higher amount of smaller water droplets. 

**Figure 7:**
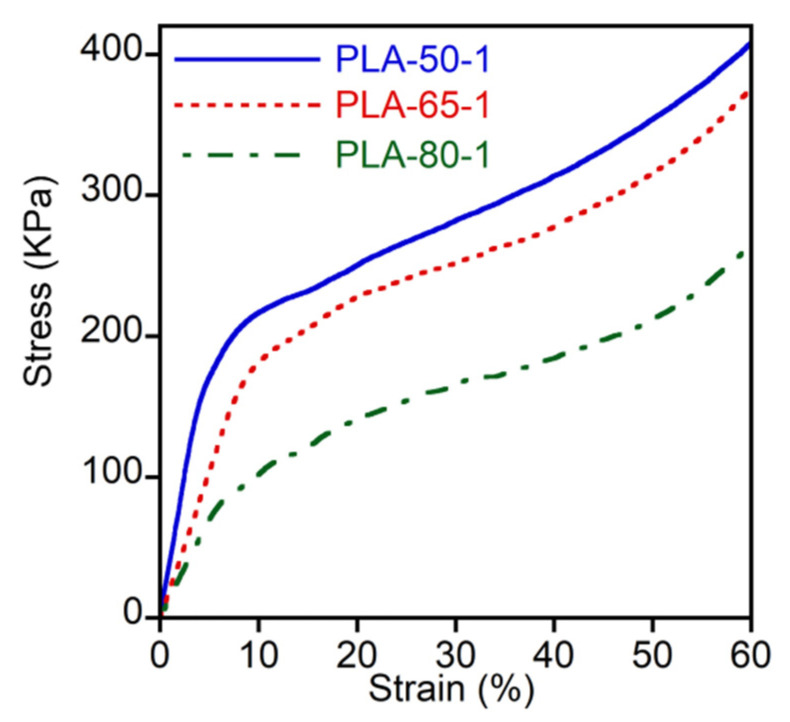
Stress–strain curves of PLA-50-1, PLA-65-1, and PLA-80-1 scaffolds recorded during compression at a strain rate of 5 mm min^−1^.

The mechanical properties of the scaffolds were evaluated by stress–strain compression tests; three selected experiments are reported in Figure 7.

**Figure 8:**
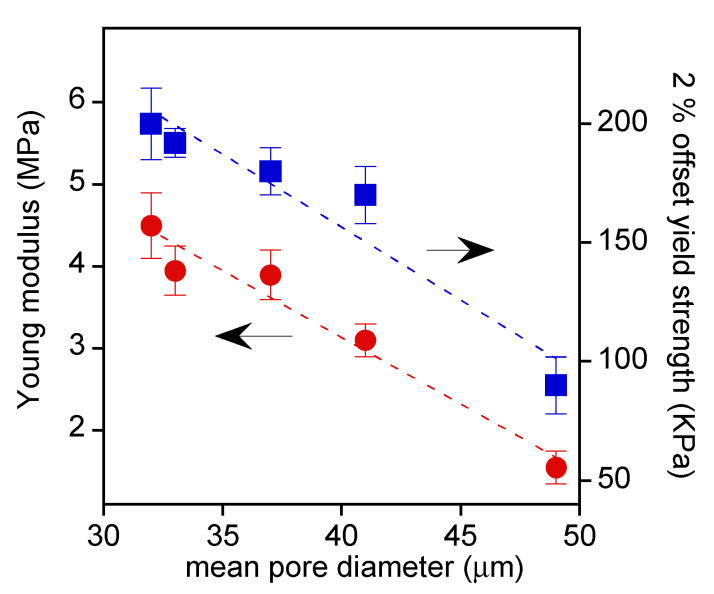
Compression Young’s modulus (red marks) and yield strength at 2% strain offset (blue marks) as a function of scaffold mean pore size.

At low strain, the stress increased linearly up to the nonreversible sample deformation in correspondence to the curve bending. The Young’s modulus, evaluated from the slope of the initial linear region, and the 2% offset yield strength are reported in Figure 8 as a function of the mean pore dimension, which is the feature that mainly influenced the scaffolds’ mechanical properties.

In fact, the figure shows that by decreasing the pore dimension, the scaffold rigidity and toughness were increased.

The yield point was not very defined, since the scaffolds were flexible under compression and did not break apart. However, the yield strain was located at about 5–8%, and did not show dependence on sample morphology or preparation conditions.

**Figure 9:**
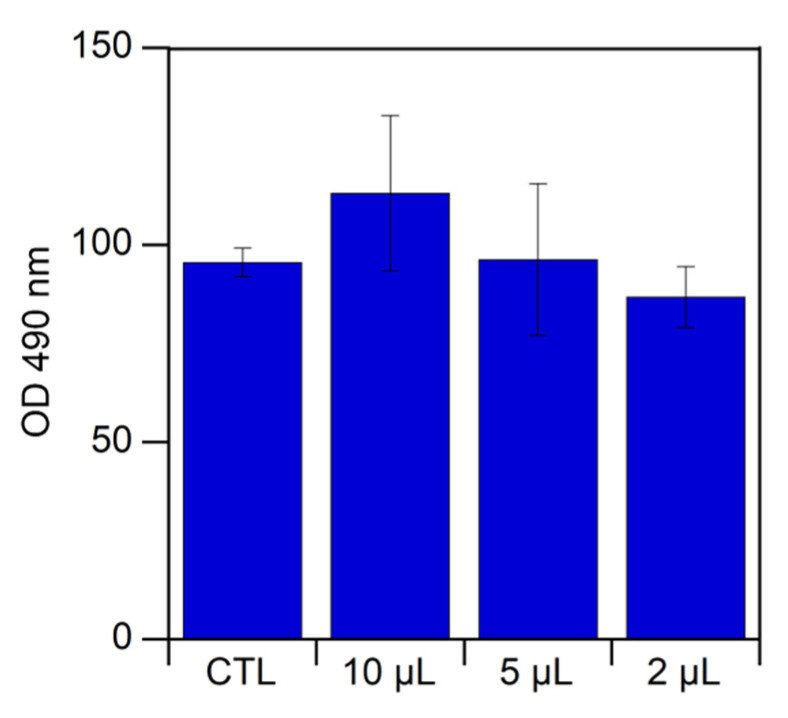
Chondrocyte viability assessed by MTS method. Cells were cultivated for 48 h in presence of 2 μL, 5 μL, and 10 μL of [Ch][Tau]. Untreated cells were considered the control (CTL), which is reported as 100%. Results represent the mean ± S.D. of data obtained by three different experiments.

To establish if [Ch][Tau] was devoid of any toxicity, human primary a (HPCs) were cultured in the presence of different amounts of ionic liquid (2 μL, 5 μL, and 10 μL). The results of an MTS assay, which was carried out after 48 h of incubation, are reported in Figure 9. 

As can be seen, up to the tested maximum salt concentration, [Ch][Tau] did not show any toxic effect. In fact, the cell viability measured after 48 h for the ionic liquid was comparable to that of the control.

## 4. Discussion

In addition to the characteristics necessary in the manufacturing process, the choice of suitable solvents and chemicals for the preparation of polymer-based items in contact with cells or tissues, including carriers for drug delivery or scaffolds for tissue engineering, cannot prescind their safety. In this context, the present research aimed at finding a facile method to prepare scaffolds for tissue engineering by using polylactides, nontoxic green solvents, and chemicals. The chosen polymer solvent, ethyl acetate, is considered one of the organic liquids with less toxicity and of lower risk to human health, and belongs to Class 3, which includes solvents known as a human health hazards at levels normally accepted in pharmaceuticals [38]. Differently, chloroform or tetrahydrofuran, commonly used to dissolve PLA in different preparations, are in Class 2, which includes solvents that should be limited in pharmaceutical products due to their inherent toxicity. Furthermore, EtOAc has a low burden for the environment [39]. 

Due to the lack of solubility of taurine in organic media and the formation of zwitterion, which could depress the amino group reactivity, the use of a suitable taurine salt allows the aminolysis reaction in water and alcohols, such as methanol and ethanol. As previously evidenced, the use of TBA as a counter anion could negatively impact the biocompatibility of aminolyzed products, and thus the ion exchange step has been mandatory [32]. According to these considerations, our efforts were focused not only on the solubilization of PLA in a safe solvent, but also on the use of a nontoxic taurine derivative. Hence, choline was inferred to be a good candidate for the preparation of a taurine-based ionic liquid, in terms of solubility in water and alcohols, reactivity, biocompatibility, and low environmental impact. Those features are deemed very important in the light of possible use of ionic liquids in many technological fields. Due to their key features, including low vapor tension, thermal and chemical stability, solvation properties, and recyclability, ionic liquids have been widely used for the synthesis and modification of polymers, and as catalysts, absorbents, lubricants, or surface-treatment agents [33,34,40]. Since the choice of the counter anion could deeply influence the nature of the liquid ionic, several taurine derivatives could be designed for the aminolysis of PLAs. However, in the present research, one of the main guideline was the identification of safe reactants. Although choline and taurine are molecules produced by mammalians that are largely used as supplements in human food and drinks, as well as animal feed additives [41,42], the toxicity of choline taurinate was evaluated as a precautionary measure. The experiments were carried out by using chondrocytes, a cell typology that, according to the found scaffold properties, could be seeded on the scaffolds. The results showed that [Ch][Tau] had no negative effect on these cells. The ionic liquid was used to react with PLA bond via aminolysis, producing oligomers endowed with a hydrophobic polyester tail and a hydrophilic head, and thus surfactant activity. This allowed the preparation of a stable emulsion composed of a continuous solution phase of polymer in EtOAc and of water droplets as a dispersed phase, which acted as a porogen agent. A solid scaffold was obtained by drying the emulsion without the freezing process. The prepared constructs showed interconnected pores and a porosity nearly equal to that expected from the volumes of organic and water phases. This occurred because the polymer solution obtained at 70 °C formed a stable physical gel at room temperature that was not subjected to a large shrinking upon drying. These findings allowed us to foresee the possibility to modulate the porosity by varying the volume ratio between the two phases.

Another advantage of the proposed methods was the introduction of the sulfonic group of Tau onto the PLA scaffold. In addition, to increase the hydrophilicity of the polyester, which is notoriously very low, the presence of SO_3_^−^, in anionic form and at low pH, favored the electrostatic interaction with positively charged bioactive molecules, such as collagen [32]. Indeed, it is well known that the absorption of this protein improves the cell-surface recognition and adhesion.

## 5. Conclusions

Emulsion drying is a widely employed procedure to obtain porous scaffolds, although it can show some drawbacks related to the use of surfactants and, according to the employed polymer matrix, nonsafe solvents. As far as PLAs, one of the most studied materials for biomedical application due to its very appealing properties, they suffer from low hydrophilicity, solubility in chlorinate hydrocarbons, and lack of functional groups able to modify their surface chemistry. In this paper, a scaffold preparation procedure that could overcome these problems was designed and applied. The key advantages found were:

- Use of nontoxic solvents and reactants;
- Simple one-pot preparation;
- No scaffold shrinking upon drying;
- High, and possibly adjustable, porosity;
- Tunable pore dimensions, and then, mechanical properties;
- Grafting of sulfonic groups able to interact electrostatically with collagen at low pH.

According to the observed chemical, morphological, and mechanical properties of the samples, as well as the previous findings regarding the enhanced chondrocytes’ adhesion on a PLLA surface modified by a taurine–collagen complex, the described scaffolds could be suitable for the growth of medium-stiffness tissues, such as cartilage.

## Figures and Tables

**Table 1:** Sample code, PLLA weight fraction in PLLA+PDLLA mixtures, and [Ch][Tau] solution concentrations used for scaffold preparation; mean pore dimension evaluated by SEM analysis; total (*p_t_*); and open porosity (*p_o_*) of the scaffolds.

Sample Code	PLLA/(PLLA + PDLLA) (wt %)	[Ch][Tau] in water (wt/v %)	Mean Pore Dimension (μm)	*p_t_* (v %)	*p_o_* (v %)
PLA-100-1	100	1			
PLA-80-1	80	1	49 ± 4	95	94
PLA-65-0.32	65	0.32	37 ± 3		
PLA-65-1		1	41 ± 4		
PLA-65-5		5	32 ± 3	95	93
PLA-50-1	50	1	33 ± 3	95	92

## Abbreviations

ATR-FTIR	Attenuated total reflection–Fourier-transform infrared spectroscopy
Ch	Choline
ChOH	Choline hydroxide
[Ch][Tau]	Choline taurinate
EtOAc	Ethyl acetate
HPCs	Human primary chondrocytes
MTS	3-(4,5-dimethylthiazol-2-yl)-5-(3-carboxymethoxyphenyl)-2-(4-sulfophenyl)-2H-tetrazolium
*p_o_*	Open porosity
*p_t_*	Total porosity
PLA	Polylactides or polylactic acids
PDLA	Poly(*D*-lactide)
PDLLA	Poly(*DL*-lactide
PLLA	Poly(*L*-lactide)
S.D.	Standard deviation
Tau	Taurine
TBA	Tetrabutylammonium
*V_l_*	Pore volume
*V_sc_*	Scaffold geometric volume
XPS	X-ray photoelectron spectroscopy
*ρ_PLA_*	PLA density
*ρ_sc_*	Apparent scaffold density
*σ*	Apparent stress
*ε*	Strain
*ϑ_r_*	Receding contact angle
*ϑ_a_*	Advancing contact angle
